# Glioma and the gut–brain axis: opportunities and future perspectives

**DOI:** 10.1093/noajnl/vdac054

**Published:** 2022-04-14

**Authors:** Antonio Dono, Jack Nickles, Ana G Rodriguez-Armendariz, Braden C McFarland, Nadim J Ajami, Leomar Y Ballester, Jennifer A Wargo, Yoshua Esquenazi

**Affiliations:** 1 Vivian L. Smith Department of Neurosurgery, The University of Texas Health Science Center at Houston, Houston, Texas, USA; 2 Department of Pathology and Laboratory Medicine, The University of Texas Health Science Center at Houston, Houston, Texas, USA; 3 McGovern Medical School and Center of Precision Health, School of Biomedical Informatics, The University of Texas Health Science Center at Houston, Houston, Texas, USA; 4 Northeastern University, College of Science, Boston, Massachusetts, USA; 5 Memorial Hermann Hospital, Houston, Texas, USA; 6 Tufts Medical Center, Boston, Massachusetts, USA; 7 Escuela de Medicina y Ciencias de la Salud, Tecnológico de Monterrey, Monterrey, Nuevo Leon, Mexico; 8 Department of Cell, Developmental and Integrative Biology, University of Alabama at Birmingham, Birmingham, Alabama, USA; 9 Department of Genomic Medicine, The University of Texas MD Anderson Cancer Center, Houston, Texas, USA; 10 Department of Pathology, The University of Texas MD Anderson Cancer Center, Houston, Texas, USA; 11 Department of Translational Molecular Pathology, The University of Texas MD Anderson Cancer Center, Houston, Texas, USA; 12 Department of Surgical Oncology, The University of Texas MD Anderson Cancer Center, Houston, Texas, USA

**Keywords:** fecal metabolites, glioblastoma, glioma, gut–brain axis, gut microbiome

## Abstract

The gut–brain axis has presented a valuable new dynamic in the treatment of cancer and central nervous system (CNS) diseases. However, little is known about the potential role of this axis in neuro-oncology. The goal of this review is to highlight potential implications of the gut–brain axis in neuro-oncology, in particular gliomas, and future areas of research. The gut–brain axis is a well-established biochemical signaling axis that has been associated with various CNS diseases. In neuro-oncology, recent studies have described gut microbiome differences in tumor-bearing mice and glioma patients compared to controls. These differences in the composition of the microbiome are expected to impact the metabolic functionality of each microbiome. The effects of antibiotics on the microbiome may affect tumor growth and modulate the immune system in tumor-bearing mice. Preliminary studies have shown that the gut microbiome might influence PD-L1 response in glioma-bearing mice, as previously observed in other non-CNS cancers. Groundbreaking studies have identified intratumoral bacterial DNA in several cancers including high-grade glioma. The gut microbiome and its manipulation represent a new and relatively unexplored area that could be utilized to enhance the effectiveness of therapy in glioma. Further mechanistic studies of this therapeutic strategy are needed to assess its clinical relevance.

**Key Points**

- There are gut microbiome differences among tumor-bearing mice/glioma patients.
- The antibiotic effects on the microbiome may affect tumor growth and modulate the immune system.
- Intratumoral bacterial DNA in several cancers including GBM has been identified.

Gliomas, a type of central nervous system (CNS) tumor, account for 80% of all malignant brain tumors and are the leading cause of death within the field of neuro-oncology.^1^ Despite the frequency of this diagnosis, glioma etiology is unclear. Although progress has been made in specific aspects of this disease, such as molecular markers that have prognostic significance, treatment strategies have been seemingly stagnant over the past decades.

Furthering research of the microbiome, which houses the commensal bacteria of the gut, has presented a valuable new dynamic in the treatment of cancer and CNS diseases.^2–10^ The microbiome can communicate with the brain via the gut–brain axis: a bidirectional feedback loop that utilizes fecal metabolites such as short-chain fatty acids (SCFAs) and neurotransmitters, among other pathways.^11^ Furthermore, recent research has illuminated the influence of the gut microbiome and bacteria-derived metabolites in the effectiveness and side effect protection of chemo-, radio-, and immunotherapies.^6,12,13^

In neuro-oncology, recent studies involving gliomas have shown that tumor growth affects the bacterial composition of the gut microbiome, fecal metabolites, and the innate immune system.^14–16^ This article reviews the literature on the gut microbiome, the gut–brain axis, and its relationship with CNS diseases and cancer, and explores the emerging evidence of the role that the gut microbiome plays in brain tumors, specifically glioma. We highlight the potential implications of the gut–brain axis in neuro-oncology and future areas of research.

## Microbiome

The microbiome has been a fascinating area of research since the ability to use next generation sequencing techniques to profile microbial communities within the body. The microbiome encompasses all the symbiotic microbes and their associated genes that have coevolved with humans. These microorganisms have created a homeostatically driven ecosystem within their host.^17^ The gut microbiome establishes early in life and varies within, and among populations, based on factors such as diet, ethnicity, and age.^17–19^ The role of diet on the gut microbiome is paramount, as diet directly influences the microbiome diversity and abundance in the gut, for example, higher fibers promote the growth of microbes specialized in the production of SCFAs like *Bifidobacterium.*^20,21^

**Figure 1.**
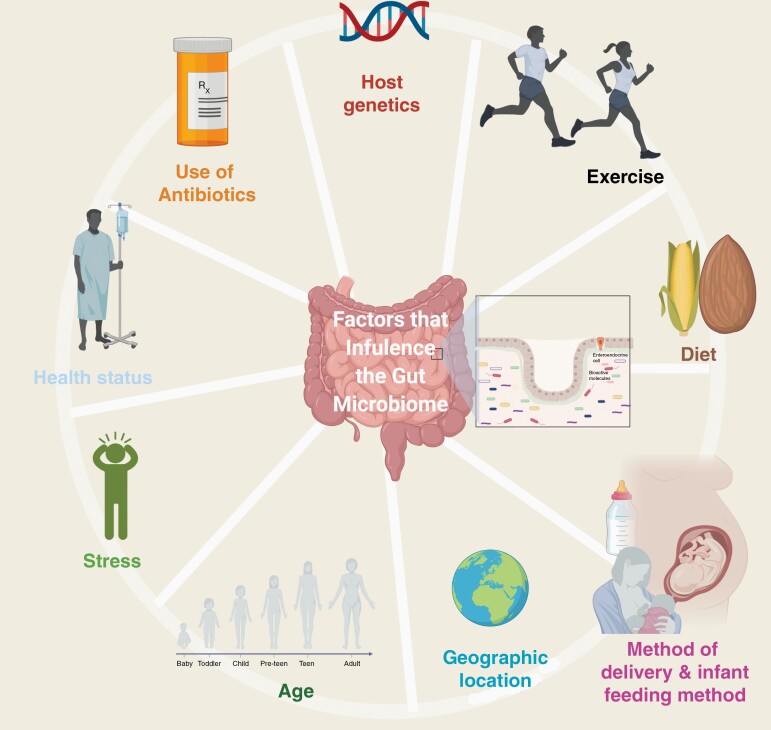
Factors known to influence the gut microbiome composition.

Small, daily fluctuations in the gut microbiome composition occur, however, larger variations arise during a lifespan (Figure 1). The microbiome changes that occur with advanced age might be connected to the declining immune system and inflammatory responses.^22^ The gut microbiome functions include: modulation of immune activation and response, epithelial barrier integrity, nutrient absorption and storage, conversion of luminal compounds to metabolites, host–bacterial interactions at the mucosal surface, and long-term modulation of behaviors and brain processes.^17,22–26^

Since the microbiome plays a critical role in normal physiological function, several studies have sought to establish the taxonomic composition and structure in healthy individuals. However, the definition of a “normal” gut microbiome remains inconclusive due to the high compositional variability in microbial taxa, even within healthy individuals and twins.^18,27,28^ Instead, a more practical approach is to look for a “functional core” in which specific properties, such as basic metabolic functions and regulatory pathways, are present and maintained.^27,29^ Large studies have shown that despite considerable interpersonal variation in microbial taxa composition, genes encoding specific metabolic functions are conserved.^17,18^ For example, the healthy gut microbiome can modulate their bacterial proportions via the host’s innate immunity, inducing the expression of specific genes that upregulate antimicrobial proteins.^24^

Dysbiosis, or a disruption in the normal functions or composition of the microbiome, is caused by a deviation from the functional core produced by factors such as antibiotics, lack of sufficient bacterial diversity, and microorganisms that react to therapies (including chemotherapy) causing toxicity or reducing pharmaceutical efficacy.^17,30,31^ Dysbiosis is an important aspect of the gut microbiome to consider, as it might potentially increase the risk of disease due to an inability to effectively respond to environmental changes.^32^ This lack of resilience leaves the host susceptible to disease. However, it is still unclear whether dysbiosis is a cause, or a response to, a particular disease state.^17,32^

## The Gut–Brain Axis

The bidirectional communication between the CNS and the enteric nervous system forms a network called the gut–brain axis.^11^ Initially, it was believed that disruptions to the gut microbiome only created pathophysiological disorders locally in the gut, as seen in irritable bowel syndrome.^26^ However, more recent research has shown pathophysiological consequences of dysbiosis, not only in the gut, but also in the CNS.^11,26,33^ A large body of preclinical research, using germ-free (GF) and wild-type mice, has substantiated evidence that an alteration in brain signaling and behavior can be caused by manipulation of the gut microbiome.^34,35^ Specific alterations include memory dysfunction following bacterial infection, reduced neurotransmitter receptor expression in GF mice, and alterations in neuron excitability after probiotic administration, among many others.^33,34,36^ The translatability of these studies is limited because, although GF mice provide an organism with no microbial influence, the conditions in which they are reared have far-reaching physiological effects. These differences make extrapolating the data to human populations more difficult.

Human trials are more complex because of interindividual microbiome differences. To circumvent this, brain imaging has been used to link microbial ecology with various neural networks.^22,26^ Manipulation of the gut microbiome using antibiotics has shown increased subcortical and frontoparietal brain connectivity as well as improved cognitive function in a small cohort of minimal hepatic encephalopathic patients.^37^ Thus, both, preclinical and clinical evidence, show the cross-communication of the gut and the brain.

The gut affects neurological function via the neuroendocrine and immunological pathways, while the brain affects the composition of the microbiome via the autonomic nervous system (ANS).^26^ With neurons located centrally and peripherally, the ANS creates a complex feedback loop between the brain and the gut. Both branches of the ANS, in coordination with the enteric nervous system, via the hypothalamic–pituitary–adrenal axis, can induce CNS modulated changes to the microbiome including gut motility, rate of intestinal transit, and mucus secretion.^22,26^ Much of this feedback loop relies upon the vagus nerve. Afferent neurons of the vagus nerve carry signals from multiple innervated layers of the gut to the nucleus tractus solitarius (NTS) of the brain. The NTS, which has numerous different neurotransmitters and neuropeptide receptors can then act as a mediary to the brain. An integrated parasympathetic response is then conducted back through the vagus nerve, causing bodily and behavioral changes.^22,38^ Sympathetic innervation, through less direct pathways, mainly functions to maintain the integrity of the intestinal mucus layer.^39^

**Figure 2.**
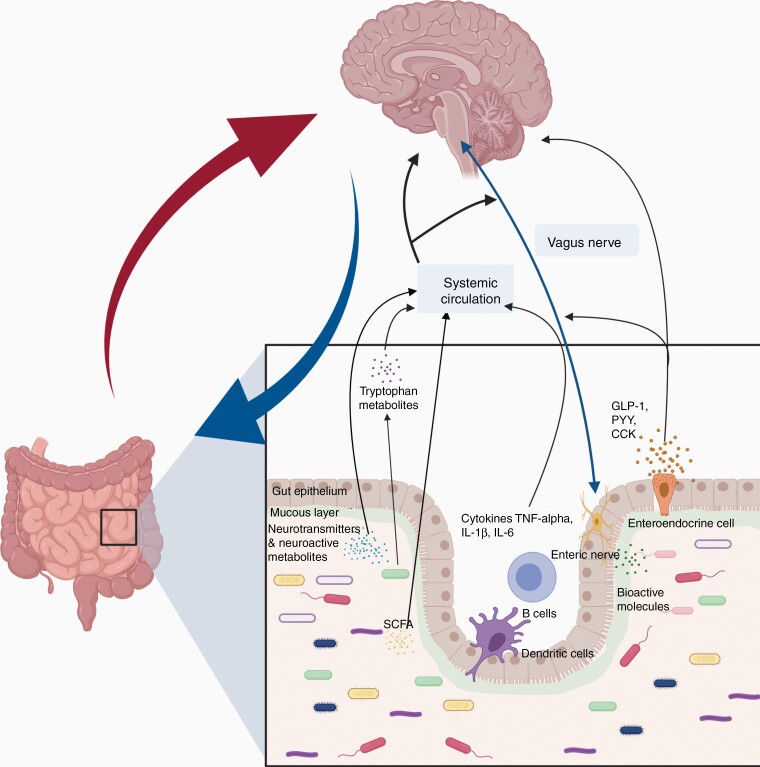
The gut–brain axis communication is bidirectional and mediated by several pathways. This communication is performed through hormonal (hypothalamic–pituitary–adrenal axis), metabolic (short-chain fatty acids and neurotransmitters), and immunologic pathways. Moreover, the gut microbiome interacts with the brain through the enteric nervous system and vagus nerve, which can be influenced either by the gut microbiota itself or indirectly through a local effect on the pathway by neuroactive compounds (eg, noradrenaline, serotonin, dopamine, tryptophan, and short-chain fatty acid).

Another mechanism by which the axis operates is through metabolites. Blood and cerebral metabolites affect neuroendocrine responsiveness. The microbiome modulates metabolite levels by synthesizing metabolic reactions.^25^ Altered levels of SCFAs have been correlated with several CNS diseases and have been found in cerebrospinal fluid (CSF) and directly in the brain.^15,22^ Furthermore, SCFAs affect the immune response in many different ways including regulation of antigen-presenting cells and manipulation of T-cell production of IL-10, TH-1, and TH-17.^40^ Another family of molecules that play a role in the gut–brain axis is the neurotransmitters. The microbiome synthesizes and responds to several neurotransmitters including serotonin, norepinephrine, and other catecholamines. CNS diseases greatly skew the number of fecal neurotransmitters outside of the normal physiological range. This local change in the gut environment then creates systemic changes globally.^15^ A summary of the gut–brain axis communication is depicted in Figure 2.

## Gut Microbiome and the Blood–Brain Barrier

The blood–brain barrier (BBB), an extension of the neural microvasculature composed of a network of endothelial cells sealed with tight junctions, contributes to maintaining CNS homeostasis.^41^ The BBB obstructs the diffusion of pathogens and hydrophilic molecules from the CSF and surrounding vasculature while permitting critical gases (O_2_, CO_2_) and lipid-soluble substances (glucose).^41^ However, the tight junctions of the BBB and their associated proteins are susceptible to degradation, which compromises its integrity.

The gut microbiome influences the development and maintenance of tight junctions of the BBB. GF mice, who lack a normal microbiome, have significantly higher levels of BBB permeability.^42^ It was observed that this is the result of reduced expression of occludin and claudin-5, key regulators of the BBB’s tight junctions.^42^ Interestingly, microbial colonization of GF mice gut has been shown to decrease permeability and increase the expression of tight junction proteins.^42^ Additionally, a study utilizing antibiotic-treated mice demonstrated that *Lactobacillus* and sodium butyrate administration reduces the permeability of the BBB in aged mice.^43^ Furthermore, gut microbial selective depletion of acetate and propionate-producing bacteria (through oral antibiotics) has been shown to increase BBB permeability in adult rhesus monkeys.^44^ This was exhibited by a reduced CSF/serum albumin ratio. Taken together these data demonstrate the important role of the gut microbiome in the BBB.

The BBB presents an obstacle to drug delivery in the brain. Although it is widely accepted that glioblastoma (GBM) patients have BBB disruption, it is now known, that all GBM patients have intact BBB tumor regions.^45^ Currently, efforts are being made to overcome the BBB to enhance drug delivery.^46^ Research is needed to further understand the relationship between the gut microbiome and the BBB, which might open novel methods to enhance drug delivery to treat CNS diseases, including gliomas.

## Gut Microbiome and CNS Diseases

Accumulating evidence from both in vivo, and clinical studies, has implicated the gut microbiome in a variety of psychiatric, neurological, and neurodegenerative diseases.^2,3,22,47–51^ The relationship between multiple sclerosis (MS) and autism spectrum disorder (ASD) and the gut microbiome has been thoroughly investigated in both animals and humans.^47,50,52,53^ In both MS and ASD, SCFAs producing bacteria and SCFAs levels have been identified to be decreased.^54,55^ In stroke animal models, several studies have shown the role of the gut microbiome in outcome through the modulation of SCFAs.^2,56^ Meanwhile, in Alzheimer’s disease, the gut microbiome has been related to β-amyloid plaques and the pathophysiology of the disease.^57^ Other interesting findings had correlated amyotrophic lateral sclerosis, epilepsy, and Parkinson’s disease outcomes due to specific microbial-derived metabolites or drug metabolism from bacteria.^3,58,59^ Even though the findings of a strong relationship between the gut microbiome and CNS diseases are paving the path in neurosciences, there is still much that remains to discover.

## Gut Microbiome and Cancer

It is estimated that microbes can be implicated in ~15%–20% of cancers.^60^ The recognition of the importance of the relationship between microbes in the gut and systemic tumors is increasing. The role of the gut microbiome in carcinogenesis,^61^ as well as, its influence in response to treatments like immunotherapy^4,5^ are interesting observations that could have a great impact in the field of oncology. Recent studies have revealed that human tumors harbor-specific bacteria that can be identified across tumor types,^62,63^ offering an interesting insight into the relationship between the microbiome and oncogenesis. However, the role of these tumor-associated bacteria is incompletely understood and more research is needed.

### The Gut Microbiome and Chemotherapy

The microbiome plays an important role in the pathophysiology of cancer, not only in its formation, but also in the efficacy of treatments. The presence of commensal bacteria modifies anticancer drugs response by modulating the immune system and microenvironment.^12,64^

Chemotherapy utilizing CpG oligonucleotide, cyclophosphamide, and platinum-based agents cause translocation of gram-positive species into secondary lymphoid organs.^12^ Once there, naive CD4^+^ T cells are differentiated into TH-17 and TH-1 cells which produce interleukin-17 and interleukin-1, respectively, upon activation.^12^ Both proinflammatory cytokines contribute to tumor suppression and eradication.^65^ GF and antibiotic-treated mice show a marked reduction of TH-17 and TH-1 cells which correlates with an inability to effectively suppress tumorigenesis.^12^ Because chemotherapy’s tumoricidal properties might be affected by commensal bacteria, researchers have speculated about the possibility to enhance chemotherapy with “immune-stimulatory organisms.”^66^ However, more research is needed to push the boundaries of the vast array of possibilities offered by microbiome modulation with regard to the prevention and treatment of cancer.

### The Gut Microbiome and Immunotherapy

The use of immunotherapy to promote antitumor immune response has revolutionized the treatment of multiple cancers, particularly melanoma, and epithelial tumors.^67–69^ Seminal studies on the gut microbiome have revealed the key role of specific gut bacteria (eg, *Ruminococcaceae* family, *Akkermansia muciniphila*, *Bifidobacterium pseudolongum*) in modulating the response to immune checkpoint inhibitors (ICIs) in both, melanoma and epithelial tumors.^4–9^ In addition, a recent study evaluating 52 patients with solid tumors treated with ICIs, identified high concentrations of some fecal (acetic acid, propionic acid, butyric acid, valeric acid) and plasma (isovaleric acid) SCFAs were associated with improved progression-free survival.^70^ The first human clinical trials exploring the safety of gut microbiome transplants in combination with ICIs for advanced melanoma have shown its safeness, long-term fecal microbiota transplant engraftment, and the response to anti-PD-1 therapy in a subset of previously PD-1-refractory cases.^71,72^ Despite these exciting advancements in melanoma and other cancer, how the microbiome might influence immunotherapy in glioma is an important but unanswered question that might identify patients that will respond to this therapy.

## Gut Microbiome and Glioma

**Figure 3.**
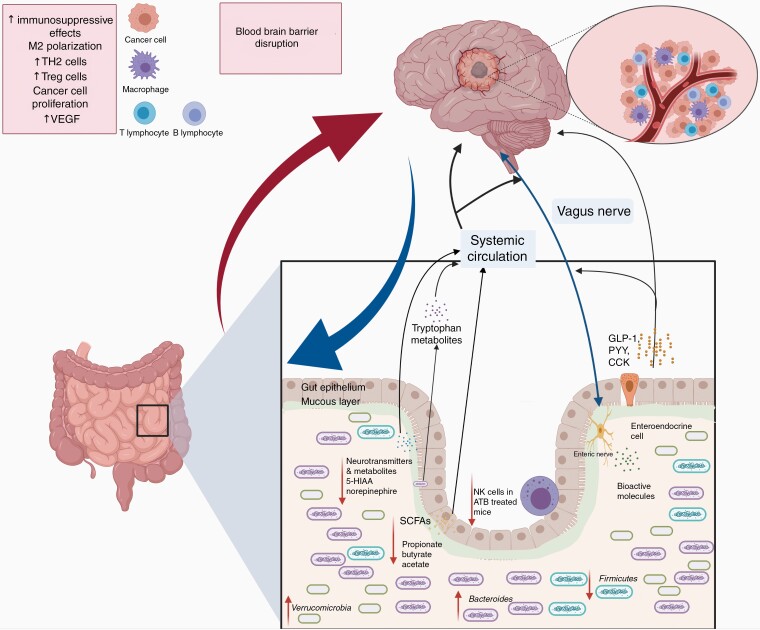
Glioma alters the gut microbiome, fecal metabolites, and immune system. Illustrative figure derived from animal model data. Tumor growth with consequent blood–brain barrier disruption changes the relative taxa abundance (decrease in *Firmicutes* phylum and increase in *Verrucomicrobiota* and *Bacteroidetes* phyla) in mice. Similar results were observed in humans compared to the control in the same study. Glioma-bearing mice have been identified to present a decrease in short-chain fatty acids and important neurotransmitters (5-hydroxindoloacetic acid and norepinephrine). Moreover, it has been shown that antibiotic treatment induces NK cell impairment in glioma-bearing mice.

The relationship between the gut microbiome and glioma is a new and active field of investigation that might open therapeutic venues to a disease in which current treatment efficacy is limited. As observed in other CNS diseases and cancer, glioma mice models have shown that tumor development alters the gut microbiome prior to weight loss.^14^ Glioma-bearing mice present gut dysbiosis—measured by changes in the *Firmicutes*:*Bacteroidetes* ratio (F:B). In addition, to changes in *Firmicutes* and *Bacteroidetes* phyla, an increase in the relative abundance of the *Verrucomicrobia* phylum driven by *Akkermansia*, was observed. Interestingly, temozolomide (TMZ) administration hampered the changes observed in glioma-bearing mice.^14^ This study also demonstrated that the mice results translate to humans, as fecal samples derived from glioma patients compared to healthy controls showed similar taxa changes.^14^ A study evaluating fecal metabolite changes after tumor development demonstrated that the most important SCFAs (butyrate, acetate, propionate) and important neurotransmitters (norepinephrine, 5-hydroxyindoleacetic acid [5-HIAA]) were diminished after tumor development. The norepinephrine and 5-HIAA decreased levels were also identified in a small cohort of glioma patients compared to household member controls.^15^ In addition, a recent study by D’Alessandro et al.^16^ showed that glioma-bearing mice treated with antibiotics have increased tumor growth, changes in natural killer cells, and changes in microglia phenotype.^16,73^ Altogether, these studies demonstrate the interplay between the gut–brain axis in glioma mice models and humans (Figure 3). Moreover, these studies support the possible role of the gut microbiome in the modulation of brain tumor immunosuppression. However, studies evaluating the mechanisms in which the gut microbiome might provoke changes in the tumor microenvironment are needed (Table 1).

**Table 1.** Summary of studies evaluating the role of the gut microbiome in glioma

Study	Study subjects	Summary findings
D’Alessandro et al.^16^	Glioma-bearing mice	Antibiotic treatment of glioma-bearing mice promoted tumor growth and changed the NK cell subsets and microglia phenotype.
Dono et al.^15^	Glioma-bearing mice and glioma patients	Norepinephrine and 5-HIAA were decreased in mice and humans with glioma, compared to control. Additionally, glioma-bearing mice had decreased levels of SCFAs.
Patrizz et al.^14^	Glioma-bearing mice and glioma patients	An increased in *Verrucomicrobiota* and *Bacteroidetes* and decrease in *Firmicutes* was observed in mice and humans. In mice, TMZ administration abrogated the microbial taxa changes.
Dees et al.^76^	Humanized glioma-bearing mice	Humanized gut microbiome mouse models responded differently to PD-1 inhibitors. Taxa comparison between glioma-bearing mice that responded to anti-PD-1 revealed high abundance of *Bacteroides cellulosilyticus*.

5-HIAA, 5-hydroxyindoleacetic acid; NK, natural killer cells; PD-1, programmed cell death protein 1; SCFA, short-chain fatty acids; TMZ, temozolomide.

### The Gut Microbiome and TMZ

TMZ, part of the standard of care treatment for GBM, is a unique pharmacological oral agent. Studies have previously assessed the drug–microbiota interactions in chemotherapeutic agents.^74^ Cyclophosphamide (another alkylating agent) anticancer immune effects are modulated by the gut microbiome.^12^ A recent study evaluating the effects of TMZ on the gut microbiome identified that mice treated with serial oral TMZ gavage had significant changes in beta diversity (a measure of similarity or dissimilarity between 2 groups), *Firmicutes*:*Bacteroidetes* ratio (a ratio commonly utilized to describe dysbiosis, as it assesses the 2 most important microbiome phyla), *Muribaculaceae* and *Ruminococcaceae* families. However, these changes were not observed in a small cohort of glioma patients after TMZ and radiotherapy.^14^ Larger cohort studies are needed to evaluate if TMZ changes the gut microbiome of humans, as observed in mice.

### The Gut Microbiome and Glioma Immunotherapy

Recent studies demonstrate how the composition of the gut microbiome in cancer patients influences the response to ICI.^4,5^ Importantly, the abundance of *A. muciniphila* has been correlated with clinical response to PD-1/PD-L1 therapy in patients with epithelial tumors.^5^ Furthermore, fecal transplantation from ICI responders into GF mice reversed the lack of response to PD-1 therapy associated with antibiotic-induced dysbiosis.^5^ To further validate this, mice receiving nonresponder microbiome were orally supplemented with *A. muciniphila*, acquiring sensitivity to PD-1 therapy.^5^ Recently, a study identified that bacterial-derived inosine promotes ICI effects in tumor models through TH-1 cell activation by a T-cell-specific A_2A_R in a context-dependent manner.^6^ Moreover, this study showed that *A. muciniphila* and *B. pseudolongum* promote ICIs effect utilizing the inosine-A_2A_R signaling pathway.^6^ This study provides a novel mechanistic by which the gut microbiome influence immunotherapy through a microbial–metabolite immune pathway.

Despite ICI’s success in many solid cancers and glioma preclinical models, little efficacy has been seen in GBM.^75^ Interestingly, the *Akkermansia* genus is increased in tumor-bearing mice and glioma patients compared to controls.^14^ Recent efforts have shown that individual-specific human gut microbiome influences immunotherapy response, in a humanized microbiome mouse model, utilizing healthy human donors.^76^ Further studies focusing on the influence of the gut microbiome in the treatment of glioma patients could potentially identify a subset of patients with a particular microbiome who may benefit from specific therapies, especially those involving the immune system (immunotherapy, viral therapy).

### Intratumoral Microbiome in Gliomas

Intratumoral bacteria have been reported in several tumor types, including GBM.^62^ A recent study from 2 centers, evaluating intratumoral bacteria in 40 GBM tissue samples, detected 22 bacterial taxa, after stringent criteria to eliminate contaminating signals.^77^ Additionally, a tissue microarray of 32 GBM cases demonstrated intracellular tumor bacterial lipopolysaccharide and 16S rRNA staining in several cases.^77^ Although the significance of intratumoral bacteria is unknown, studies investigating its causality or leakage from ruptured vasculature and refugee due to gliomas’ immunosuppressive microenvironment are necessary. These efforts will pave our understanding of the role of the tumor microbiome in brain tumors.

## Potential Opportunities and Future Perspectives

The gut microbiome offers interesting possibilities to enhance therapies that previously failed in gliomas (including GBM). Future studies should focus on identifying if gut microbiome signatures correlate with fecal metabolites and/or cytokines that might translate into survival differences in patients. Moreover, studies evaluating the potential of therapeutic modulation of the microbiome by probiotics/fecal transplant to enhance therapeutic options for gliomas (eg, immunotherapy, viral therapy) are needed. Lastly, recent studies showing the presence of bacteria in tumor tissue, oppose current views about tumors being sterile. Is the tumor microbiome related to glioma development? Or is this a result of leakage from the BBB disruption and refuge due to a glioma-induced immunosuppressive microenvironment? These and other questions that arose from the first’s studies evaluating glioma and the microbiome need to be addressed. Improving our understanding of the relationship between glioma and gut microbiome might help us in the treatment of this devastating disease.

## Conclusions

In this review, we highlighted the potential implications of the gut–brain axis in neuro-oncology, in particular gliomas. Studies have demonstrated gut microbiome changes in tumor-bearing mice as well as glioma patients compared to controls. These microbiome differences might translate into fecal metabolite changes. The absence of a gut microbiome enhances tumor growth and decreases the innate immune system. Moreover, the gut microbiome might be related to the PD-L1 response in tumor-bearing mice. Understanding the synergy between the microbiome, antibiotics, intratumoral bacteria, the BBB, and the tumor microenvironment may pave the way for novel therapies. Studies evaluating the potential of therapeutic modulation of the microbiome by probiotics/fecal transplant to enhance therapeutic options for gliomas are needed.
